# Adaptive initial step size selection for Simultaneous Perturbation Stochastic Approximation

**DOI:** 10.1186/s40064-016-1823-3

**Published:** 2016-02-27

**Authors:** Keiichi Ito, Tom Dhaene

**Affiliations:** Ghent University - iMinds, INTEC, Gaston Crommenlaan 8 bus 201, Ledeberg, 9050 Ghent, Belgium; Noesis Solutions, Gaston Geenslaan 11 B4, 3001 Louvain, Belgium

**Keywords:** Stochastic approximation, Optimization, Direct method, Noisy function, Parameter estimation

## Abstract

A difficulty in using Simultaneous Perturbation Stochastics Approximation (SPSA) is its performance sensitivity to the step sizes chosen at the initial stage of the iteration. If the step size is too large, the solution estimate may fail to converge. The proposed adaptive stepping method automatically reduces the initial step size of the SPSA so that reduction of the objective function value occurs more reliably. Ten mathematical functions each with three different noise levels were used to empirically show the effectiveness of the proposed idea. A parameter estimation example of a nonlinear dynamical system is also included.

## Background

Simultaneous Perturbation Stochastic Approximation (SPSA) (Spall 1992) is an optimization algorithm that uses only objective function measurements in the search of solutions. Applications of SPSA include model-free predictive control (Dong and Chen 2012a, b; Ko et al. 2008), signal timing for vehicle timing control (Spall and Chin 1997), air traffic network (Kleinman et al. 1997), and marine vessel traffic management (Burnett 2004). More applications are mentioned in the introductory article by Spall (1998b). SPSA has been used successfully in many optimization problems that have high-dimensional input parameter space and the objective value is not deterministic (SPSA 2001).

In this optimization method, the initial design parameter vector $\theta$ of *D*-dimensions is perturbed simultaneously in every dimension, i.e. by adding and subtracting a perturbation vector ${\varvec{\Delta }}$ of *D*-dimensions, thus obtaining an estimate of the gradient vector *g*. Unlike the traditional finite differencing approach, it only takes two function evaluations to obtain the estimate of the gradient. Yet, the number of iteration needed for convergence to the optimum is said to be more or less the same with Finite-Difference Stochastic Approximation (FDSA) (Kiefer and Wolfowitz 1952), which in essence is an approximate steepest-descent method that uses finite-differencing to approximate the partial derivatives along each of the *D* parameters. Thus, the number of function evaluations of SPSA is *D*-fold smaller compared to FDSA (Spall 1998b). An extension to this method exists to include second-order (Hessian) effects to accelerate convergence (Spall 2000, 2009; Zhu and Spall 2002). However, we will not treat this enhancement here.

**Fig. 1:**
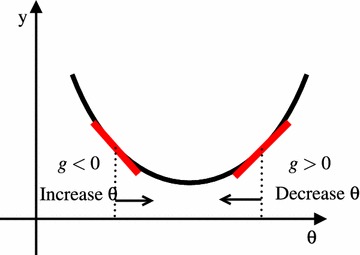
Objective value minimization using gradient descent (one variable): if gradient *g* is positive at $\theta _k$ then move to $\theta _{k+1}<\theta _k$, if gradient *g* is negative then move to $\theta _{k+1}>\theta _k$

The problem solved by SPSA in this paper can be formulated as following.

1 $$\begin{aligned} \min _{\theta \in \varTheta } f(\theta ), \end{aligned}$$

where $f(\theta )$ is the objective function and $\theta$ is a *D*-dimensional vector of parameters. We assume that each element in the vector $\theta$ is a real number and has upper and lower bounds that defines the Cartesian product domain $\varTheta$. The SPSA and FDSA procedures are in the general recursive form:

2 $$\begin{aligned} \hat{\theta }_{k+1} = \hat{\theta }_k - a_k\hat{g}_k(\hat{\theta }_k), \end{aligned}$$

where $\hat{g}_k(\hat{\theta }_k)$ is the estimate of the gradient vector $g(\hat{\theta })$ at iteration *k* based on the measurements of the objective function. The $a_k$ is the step size at iteration *k*. Equation (2) is analogous to the gradient descent algorithm in nonlinear programming, in which $g_k$ is the gradient of the objective function $\nabla {f}(\hat{\theta }_k)$. The difference is that in Eq. (2), $\hat{g}_k$ represent gradients stochastically and the effect of the noise or deviation from the true gradient is expected to cancel out as the iteration count *k* increases. The step sizes $a_k$ are normally prescribed in SPSA and FDSA as a function of *k* just like the Simulated-Annealing’s (Kirkpatrick et al. 1983) cooling schedule. This is because these methods do not assume deterministic responses in the measurements of the objective function values. Thus, unlike the nonlinear programming counterparts, adaptation of step sizes based on gradients and amount of descent achieved (such as in the line search) is usually not done in the stochastic approximation optimization methods. The rationale of the Eq. (2) is intuitively depicted in Fig. 1 for one variable case.

Under appropriate conditions, the iteration in Eq. (2) will converge to the optimum $\theta ^*$ in some stochastic sense. The hat symbol indicates an “estimate”. Thus, $\hat{\theta }_k$ denotes the estimate of the optimum $\theta ^*$ at iteration *k*. Let $y(\cdot )$ denote a measurement of the objective function $f(\cdot )$ at parameter value denoted by “$\cdot$” and $c_k$ be some small positive number. The measurements are assumed to contain some noise, i.e. $y(\cdot ) = f(\cdot ) + \text {noise}$. In SPSA, the $i\hbox {th}$ component $\hat{g}_{ki}(\hat{\theta }_k)$ of the gradient vector $\hat{g}_k(\hat{\theta }_k)$ is formed from a ratio involving the individual components in the perturbation vector and the difference in the two corresponding measurements. For two-sided simultaneous perturbations, we have

3 $$\begin{aligned} \hat{g}_{ki}(\hat{\theta }_k) = \frac{y(\hat{\theta }_k+c_k{\varvec{\Delta }}_k) -y(\hat{\theta }_k-c_k{\varvec{\Delta }}_k)}{2c_k\varDelta _{ki}}, \end{aligned}$$

where the *D*-dimensional random perturbation vector

4 $$\begin{aligned} {\varvec{\Delta }}_k = \left(\varDelta _{k0}, \varDelta _{k1},\ldots , \varDelta _{k(D-1)} \right)^T, \end{aligned}$$

follows a specific statistical distribution criterion. Here, *i* is the parameter index. A simple choice for each component of ${\varvec{\Delta }}_k$ is to use Bernoulli $\pm$1 distribution, which is essentially a random switching between +1 and $-$1. The Bernoulli distribution is proven to be an optimal distribution for the simultaneous perturbation (Sadegh and Spall 1997). Note also that in the Eq. (3), we do not evaluate $y(\hat{\theta }_k)$. The recursive equation (2) proceeds with only the responses from the two perturbed inputs $y(\hat{\theta }_k+c_k{\varvec{\Delta }}_k)$ and $y(\hat{\theta }_k-c_k{\varvec{\Delta }}_k)$.

The choice of $a_k$ and $c_k$ is critical to the performance of SPSA and suggested values can be found in Spall (1998a). At given iteration *k*:

5 $$\begin{aligned} a_k= & {} \frac{a}{(A+k+1)^\alpha }, \end{aligned}$$

6 $$\begin{aligned} c_k= & {} \frac{c}{(k+1)^\gamma }, \end{aligned}$$

where $$\alpha= 0.602$$, $$\gamma= 0.101$$, $$c\simeq \text {standard deviation of measurement noise}$$, $$A \le 10\,\% \text {of maximum number of iterations}$$, $$a= \delta \hat{\theta }_{0_{\min }} \frac{(A+1)^\alpha }{|\hat{g}_{0i}(\hat{\theta }_0)|}$$, $$k= \text {iteration index starting with 0}$$, $$\delta \hat{\theta }_{0_{\min }}= \text {smallest initial change desired in a parameter}$$.

The setting for $\alpha$ and $\gamma$ above are not optimal in the asymptotic sense, but are adapted to finite iteration settings. In practice, one of the drawbacks of SPSA is that one has to find good values for *a* and *c*, as both affect the performance of the algorithm Spall (2003, pp. 165–166) (Altaf et al. 2011; Shen et al. 2012; Radac et al. 2011; Easterling et al. 2014; Taflanidis and Beck 2008). However, for *c*, we have a tangible measure, which is the output measurement error (Spall 1998a), to select a proper value up front. If the function response is noiseless, *c* is usually not a critical parameter. On the other hand, *a* is more problematic, because no clear measure exists. It is possible to work with $\delta \hat{\theta }_{0_{\min }}$ instead of *a*, but a priori assignment of its value is still non-trivial if little is known about the function that we are trying to optimize.

A larger value of *a* generally produces better results compared to a smaller value of *a*. However, this also increases the chance that the optimization diverges to a worse solution than the starting point. Very often, the user of SPSA has to find as big *a* as possible that would not cause divergence.

To avoid divergence, an adaptation called “blocking” exists (Easterling et al. 2014; Spall 1998a) in which the objective values at $\hat{\theta }_k$ is evaluated in addition to the two perturbations. If the new objective function value is “significantly worse” than the current objective function value, the updating of $\hat{\theta }_k$ does not happen. The extra function evaluation at each iteration increases the cost of iteration by 33 %. In addition, a problem dependent threshold parameter to block the $\hat{\theta }_k$ update needs to be set up by the user.

Another way to mitigate divergence is to modify the gradient approximation $\hat{g}_k$ by “scaling” and “averaging” (Andradóttir 1996; Xu and Wu 2013). However, the methods proposed in the literature require set up of additional threshold parameters critical to their performance. Furthermore, their methods require additional gradient estimations per iteration.

Stochastic Gradient Descent (SGD) methods use noisy information of the gradient of the objective functions. On the other hand, Stochastic Approximation methods such as FDSA and SPSA only uses measurement of noisy objective values. Therefore, adaptive determination of step sizes based on (approximate) gradients and inverse Hessians in SGD literature (such as in Zeiler (2012), Bottou (2010)) may not be directly applicable to or feasible in SPSA. Convergence conditions also differ between the two. Although this does not exclude the possibility of successful import of ideas from SGD literature, in this paper, we will not delve into this direction.

This paper provides a solution to determine the appropriate values of *a* by introducing an adaptive scheme as discussed in “Adaptive initial step sizes” section. It does not require any additional objective function evaluations per iteration nor extra problem dependent parameters to set up.

## Adaptive initial step sizes

To remedy the sensitivity to *a*, we propose an adaptive stepping algorithm. At the end of each iteration *k*, we perform the adjustment described in Algorithm 1.

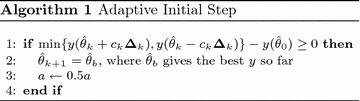



The condition requires that at least one of the two parameter perturbations produce a better (smaller) measurement of the objective function than that of initial guess of parameters $\hat{\theta }_0$ to proceed without modifying *a*. Therefore, at each iteration *k*, the smaller of the two measurements of the objective function values of perturbed parameters is compared to that of the initial value at iteration $k=0$. If the measurements of the objective values of the perturbed parameters are larger, $\hat{\theta }_k$ is reset to $\theta _b$, which is the point that gave the minimum in the history of iteration and *a* is reduced to half of its previous value. A pseudocode of the proposed SPSA with the adaptive initial step is shown in Algorithm 2. The difference between the standard SPSA and our SPSA is in line 10.

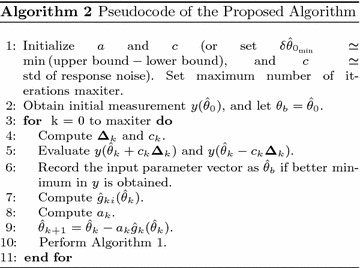



## Comments on convergence

Currently available theories of stochastic algorithms are almost all based on asymptotic properties with $k \rightarrow \infty$, and SPSA is no exception. For given conditions Spall (2003, p. 183), SPSA is proven to converge to a local optima almost surely. However, under limited function evaluation budget, we frequently encounter situations in which SPSA returns worse solution than the initial i.e. divergence. The method we propose is a practical remedy conceived in a finite *k* setting. We will show, in the next section, its effectiveness empirically via numerical experiments with *k* in the order of $10^3$.

For $\hat{\theta }_k$ to converge to the optimal solution $\theta ^*$ in *infinite* steps, the following conditions are required for $a_k$ and $c_k$ (Spall 1992): $a_k, c_k > 0$ for all *k*; $a_k, c_k \rightarrow 0$ as $k \rightarrow \infty$; $\sum _{k=0}^{\infty }a_k = \infty$, and $\sum _{k=0}^{\infty }\left( \frac{a_k}{c_k}\right) ^2 < \infty$. With Algorithm 1, $\sum _{k=0}^{\infty }a_k = \infty$ is not guaranteed. For example, if the reduction of *a* happens in every iteration *k*, the sum is convergent. In practice, the numbers of function evaluations are finite, and reductions of *a* are expected to happen only a limited number of times. Therefore, this violation is expected to pose little problem.

The intention of the proposed method is not to modify the asymptotic convergence rate of the original SPSA algorithm Spall (2003, pp. 186–188). The adaptive step takes place only if it is suspected that the objective value has become larger than at the starting point $\hat{\theta }_0$. The probability of Algorithm 1 taking place is expected to go to zero under reasonable signal-to-noise ratio as $f(\hat{\theta }_k)$ decreases. The worst situation that can happen is that the every perturbation $c_k{\varvec{\Delta }}_k$ produces worsening moves and no improvement is obtained compared to the starting point $\theta _0$. In “Computational results” section, we will confirm empirically what we have described about the convergence in finite *k* settings ($k \sim 10^3$).

Another reason to take the objective value at the starting point as the threshold value to judge divergence is that if we update this value with $y(\hat{\theta _k})$, where $k>0$, we may risk picking a point that is too low due to the noise incurred in the measurement *y*. This in turn inhibits further improvement of $\hat{\theta _k}$ for lower objective values.

In the following section, the smallest output of mathematical functions will be sought using the standard SPSA and our adaptive initial stepping SPSA. This will show the sensitivity of the function value in the final iteration to the initial step size $\delta \hat{\theta }_{0_{\min }}$ and so the sensitivity to *a*, and how the adaptive initial stepping substantially mitigates the difficulty to find the proper initial perturbation magnitude.

## Computational results

In this section, we will compare the original SPSA and our modified SPSA as described in Algorithm 2 using 10 analytical test functions and a parameter estimation example of a nonlinear dynamic system.

### Test functions

To see the effect of the new adaptive stepping algorithm in SPSA, the minimum points of ten different mathematical test functions were sought. Except for Griewank function, the following conditions were applied. The functions’ responses were minimized from arbitrary starting points $\hat{\theta }_0 \in [-2, 2]^{D}$ (*D*-dimensional product space with lower bound -2 and upper bound 2). If $\hat{\theta }_{k}=[\hat{\theta }_{k0},\hat{\theta }_{k1},\cdots ,\hat{\theta }_{ki},\cdots , \hat{\theta }_{k(D-1)}]^T$ exceeded $[-10,\ 10]$ in any of its *D* dimensions, that parameter was replaced by -10 if it was less than -10 or was replaced by 10 if it was larger than 10. For Griewank function, it was randomly started from $\hat{\theta }_0 \in [-120,\ 120]^{D}$. If $\hat{\theta }_k$ exceeded $[-600, 600]$ in any of its *D* dimensions, that parameter was replaced by −600 if it was less than −600 or was replaced by 600 if it was larger than 600. For all ten functions, the iteration was stopped when 2000 evaluations of the objective function were reached. For convenience, we will label our proposed algorithm as “A_SPSA” and the standard SPSA as “SPSA”.

The optimizations for each of the ten objective functions were started from 20 different starting points. After the 2000 iterations, the distributions of objective values were plotted with respect to $\delta \hat{\theta }_{0_{\min }}$. Eleven different values of $\delta \hat{\theta }_{0_{\min }}$ between $1.0 \times 10^{-4}$ and $1.0 \times 10^1$ (up to $1.0\times 10^2$ for Griewank) were used to make the plot. The dimensions of the functions were set to be 20, i.e. $D=20$.

The definitions of the ten functions are given in the following. The Rosenbrock function is described as

7 $$\begin{aligned} & f(\theta )= \sum _{i=0}^{D-2}{ \left (100(\theta _{i+1}-\theta _i^2)^2+(\theta _i-1)^2\right) }, \\ &\qquad i = 0,1,\ldots ,D-1,\quad D>1, \\ & \qquad f(\theta ^*) = 0, \quad \theta _i^* = 1. \end{aligned}$$

The Sphere function is described as

8 $$\begin{aligned} & f(\theta ) = \sum _{i=0}^{D-1}{\theta _i^2}, \\ & \qquad i = 0,1,\ldots ,D-1, \\ & \qquad f(\theta ^*) = 0,\quad \theta _i^* = 0. \end{aligned}$$

The Schwefel function is described as

9 $$\begin{aligned} & f(\theta )= \sum _{j=0}^{D-1}{\left( \sum _{i=0}^j{\theta _i}\right) }^2, \\ &\qquad i = 0,1,\ldots ,D-1, \\ & \qquad f(\theta ^*) = 0, \quad \theta _i^* = 0. \end{aligned}$$

The Rastrigin function is described as

10 $$\begin{aligned} & f(\theta ) = \sum _{i=0}^{D-1}{\left( \theta _i^2-10\cos (2\pi \theta_i)+10 \right) }, \\ & \qquad i = 0,1,\ldots ,D-1, \\ & \qquad f(\theta ^*)= 0, \quad \theta _i^* = 0. \end{aligned}$$

The Skewed Quartic function Spall (2003, ex. 6.6) is described as

11 $$\begin{aligned} & f(\theta ) = ({\mathbf {B}}\theta )^T{\mathbf {B}}\theta +0.1\sum _{i=0}^{D-1}{({\mathbf {B}}\theta )_i^3}+0.01\sum _{i=0}^{D-1}{({\mathbf {B}}\theta )_i^4}, \\ &\qquad i = 0,1,\ldots ,D-1, \\ & \qquad f(\theta ^*) = 0, \quad \theta _i^* = 0. \end{aligned}$$

where the matrix ${\mathbf {B}}$ in the Skewed Quartic function is a square matrix with upper triangular elements set to 1 and the lower triangular elements set to zero. The Griewank function is described as

12 $$\begin{aligned} & f(\theta ) = 1 + \sum _{i=0}^{D-1}\frac{\theta _i^2}{4000} - \prod _{i=0}^{D-1}\cos (\frac{\theta _i}{\sqrt{i}}), \\ & \qquad i = 0,1,\ldots ,D-1, \\ & \qquad f(\theta ^*) = 0,\quad \theta _i^* = 0. \end{aligned}$$

The Ackley function is described as

13 $$\begin{aligned}& f(\theta ) = -20\exp \left( -0.2 \sqrt{\frac{1}{D}\sum _{i=0}^{D-1}{\theta _i^2}}\right) \\ &\qquad -\exp \left( \frac{1}{D}\sum _{i=0}^{D-1}{\cos (2\pi \theta _i)}\right) \\ & \qquad+20-\exp (1), \\ & \quad \qquad i = 0,1,\ldots ,D-1, \\ & \quad \qquad f(\theta ^*) = 0,\quad \theta _i^* = 0. \end{aligned}$$

The Manevich function is described as

14 $$\begin{aligned} & f(\theta ) = \sum _{i=0}^{D-1}{\left[ \left( 1-\theta _i\right) ^2/2^j\right] }, \\ & \qquad i = 0,1,\ldots ,D-1, \\ & \qquad f(\theta ^*) = 0, \quad \theta _i^* = 1. \end{aligned}$$

The Ellipsoid function is described as

15 $$\begin{aligned} & f(\theta ) = \sum _{i=0}^{D-1}i\theta _i^2, \\ & \qquad i = 0,1,\ldots ,D-1, \\ & \qquad f(\theta ^*) = 0,\quad \theta _i^* = 0. \end{aligned}$$

The Rotated Ellipsoid function is described as

16 $$\begin{aligned} & f(\theta ) = \sum _{i=0}^{D-1}{\left( \sum _{j=0}^{i}{\theta _j^2}\right) ^2}, \\ & \qquad i = 0,1,\ldots ,D-1, \\ & \qquad f(\theta ^*) = 0,\quad \theta _i^* = 0. \end{aligned}$$

Each of Figs. 2, 3, 4, 5, 6, 7, 8, 9, 10 and 11 show three different cases of noisy measurements of the outputs. The subfigures (a) have no noise added, subfigures (b) and (c) have Gaussian noise added to the true output with standard deviation $\sigma$ of 0.1 and 1.0 respectively. In all the three noise levels of the ten functions, $c=0.2$ was used.

A general trend observed from the figures is that when the initial step size is large, the original SPSA tends to diverge to big objective values. The SPSA with the proposed initial step size reduction, on the other hand, effectively mitigates this divergence problem producing smaller objective values in general as the (a priori) initial step size is increased. This is because if the two function evaluations in the iteration are not smaller than the starting point value $f(\hat{\theta _0})$, the algorithm will reduce the step size (by halving *a*) and restart at $\hat{\theta _b}$, which is the point that gave the smallest output in the history of iterations. However, note that the iteration index *k* in $a_k$ and $c_k$ is not reinitialized. For the ten functions tested, A_SPSA achieved its best performance when $\delta \hat{\theta }_{0_{\min }}$ was close to 10 or 100 for Griewank function. This indicates that one can simply set the minimum perturbation $\delta \hat{\theta }_{0_{\min }}$ close to the magnitude of the difference between upper and lower bound of the parameter in consideration. This may not be a guarantee for the best results but doing so does not cause the optimization to diverge to large responses and the results achieved are not substantially worse than the cases with best settings for *a*.

As mentioned earlier, the value for *c* is important when the measurements of *y* contain noise. Figure 12 shows how the choice of *c* affects the outcome of optimizations. The figure shows the case of the 20 dimensional Sphere Function with Gaussian noise having standard deviation $\sigma = 0.1$. Among the three values of *c*, namely 0.01, 0.1 and 1.0, $c = \sigma = 0.1$ gave the best results for A_SPSA. At $c = 1.0$, however, A_SPSA showed little improvement in the objective value regardless of $\delta \hat{\theta }_{0_{\min }}$ magnitude. This is caused by *a* becoming prematurely too small in the divergent early iterations. On the other hand, the standard SPSA showed a good reduction at $\log _{10}(\delta \hat{\theta }_{0_{\min }}) = -2.0$, and $-1.5$. at both $c = 0.1$ and 1.0. This implies that for A_SPSA, a range of values of good *c* can be narrower than that of the standard SPSA. On the other hand, the choice of $\delta \hat{\theta }_{0_{\min }}$ (and therefore *a*) is much easier for A_SPSA. We can, for example, let $\delta \hat{\theta }_{0_{\min }} \simeq \min$(*U* − *L*), where min(*U* − *L*) is the minimum difference between upper and lower bounds of the domain of parameter vector $\theta$. In practice, it is better to scale all the input dimensions to fall in similar or equal intervals.

Figure 13 shows the results of optimizing the Rosenbrock and Rastrigin functions using three different values of multiplication factor of *a*: 0.1, 0.5, and 0.9. The difference in multiplication factor does not change the general trend that larger $\delta \hat{\theta }_{0_{\min }}$ produces better results and that divergence does not occur. One could tune the value of the multiplication factor, but the default value of 0.5 that we showed in the Algorithm 1 generally produces satisfactory results compared to other values of multiplication factors between 0 and 1. The Fig. 13 (b) also shows that $\delta \hat{\theta }_{0_{\min }}\simeq \min (U - L)$ may not be an optimal setting since smaller value $\delta \hat{\theta }_{0_{\min }}\simeq 10^{-1.5}$ is shown to produce better optimization results when the reduction rate is slow at 0.9. This implies that in a bumpy (highly multimodal) function like Rastrigin, the slow decrease in *a* can adversely affect the minimization of the objective value by a large number of resets to $\theta _b$. The opposite is true with Rosenbrock function in (a), in which the slow reduction factor 0.9 gave the best result at $\delta \hat{\theta }_{0_{\min }}\simeq 10^{1}$.

**Fig. 2:**
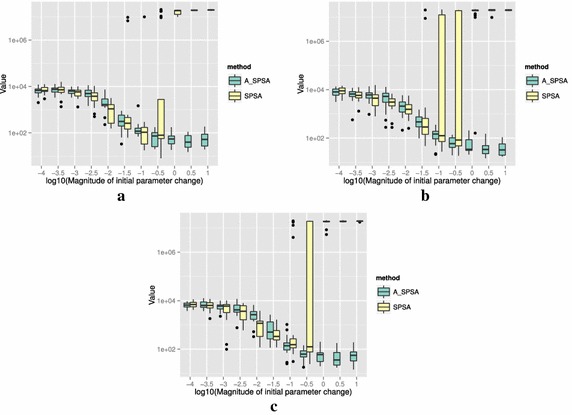
Initial parameter change $\delta \hat{\theta }_{0_{\min }}$ and distribution of responses after 2000 function evaluations for “Rosenbrock”. **a** No noise, **b** σ = 0.10, **c** σ = 1.0

**Fig. 3:**
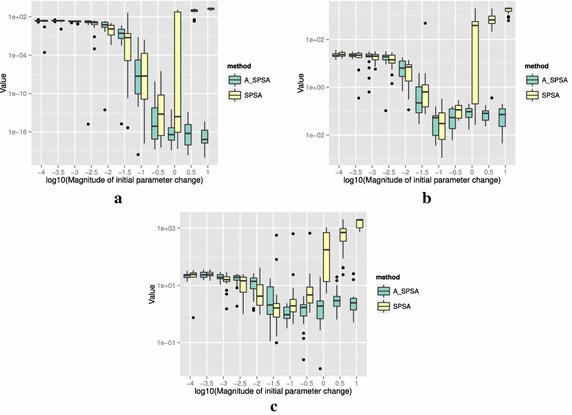
Initial parameter change $\delta \hat{\theta }_{0_{\min }}$ and distribution of responses after 2000 function evaluations for “Sphere”. **a** No noise, **b** σ = 0.10, **c** σ = 1.0

**Fig. 4:**
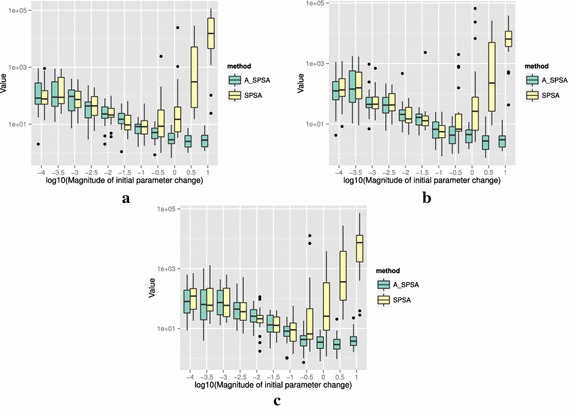
Initial parameter change $\delta \hat{\theta }_{0_{\min }}$ and distribution of responses after 2000 function evaluations for “Schwefel”. **a** No noise, **b** σ = 0.10, **c** σ = 1.0

**Fig. 5:**
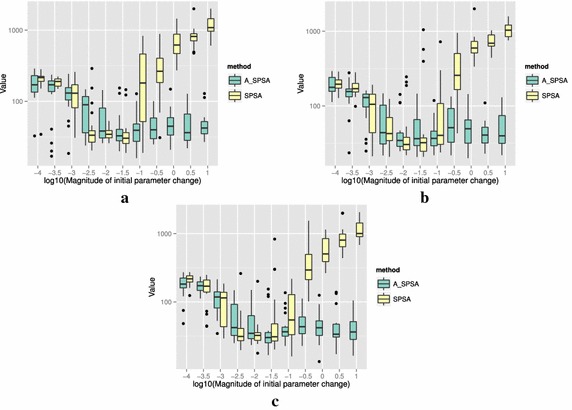
Initial parameter change $\delta \hat{\theta }_{0_{\min }}$ and distribution of responses after 2000 function evaluations for “Rastrigin”. **a** No noise, **b** σ = 0.10, **c** σ = 1.0

**Fig. 6:**
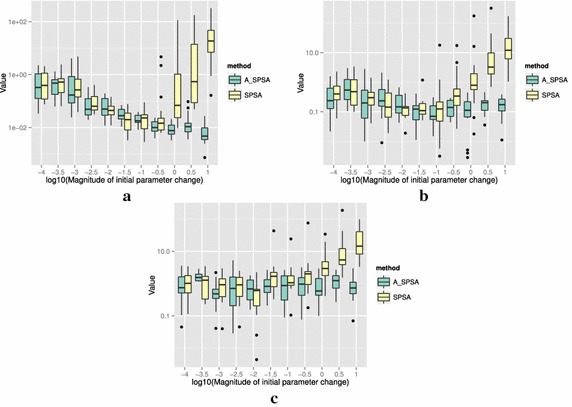
Initial parameter change $\delta \hat{\theta }_{0_{\min }}$ and distribution of responses after 2000 function evaluations for “Skewed Quartic”. **a** No noise, **b** σ = 0.10, **c** σ = 1.0

**Fig. 7:**
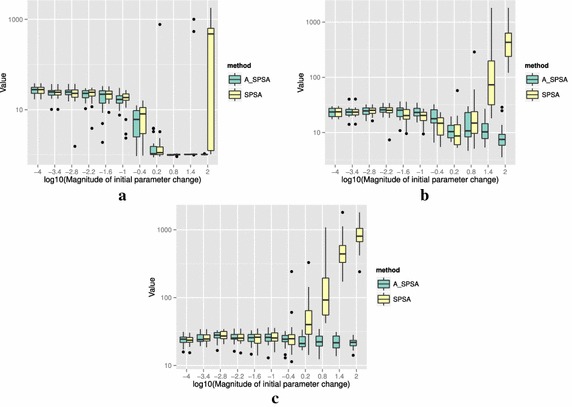
Initial parameter change $\delta \hat{\theta }_{0_{\min }}$ and distribution of responses after 2000 function evaluations for “Griewank”. **a** No noise, **b** σ = 0.10, **c** σ = 1.0

**Fig. 8:**
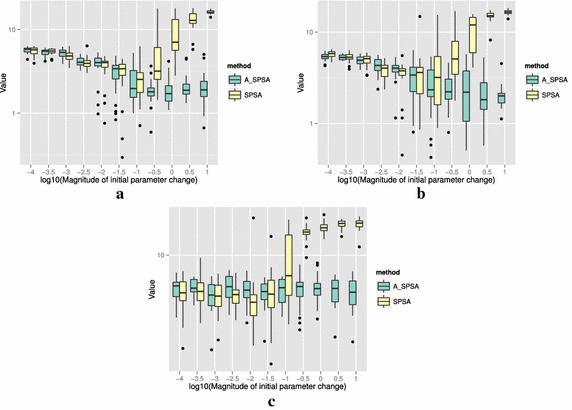
Initial parameter change $\delta \hat{\theta }_{0_{\min }}$ and distribution of responses after 2000 function evaluations for “Ackley”. **a** No noise, **b** σ = 0.10, **c** σ = 1.0

**Fig. 9:**
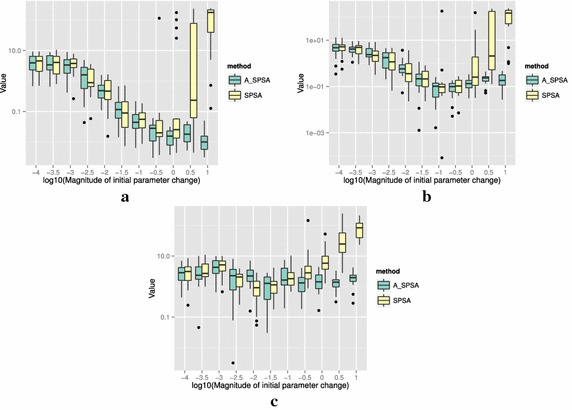
Initial parameter change $\delta \hat{\theta }_{0_{\min }}$ and distribution of responses after 2000 function evaluations for “Manevich”. **a** No noise, **b** σ = 0.10, **c** σ = 1.0

**Fig. 10:**
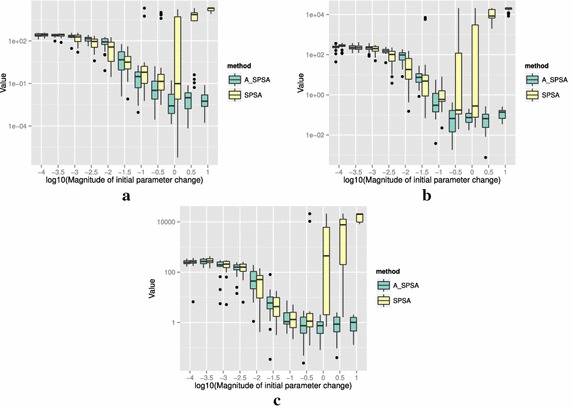
Initial parameter change $\delta \hat{\theta }_{0_{\min }}$ and distribution of responses after 2000 function evaluations for “Ellipsoid”. **a** No noise, **b** σ = 0.10, **c** σ = 1.0

**Fig. 11:**
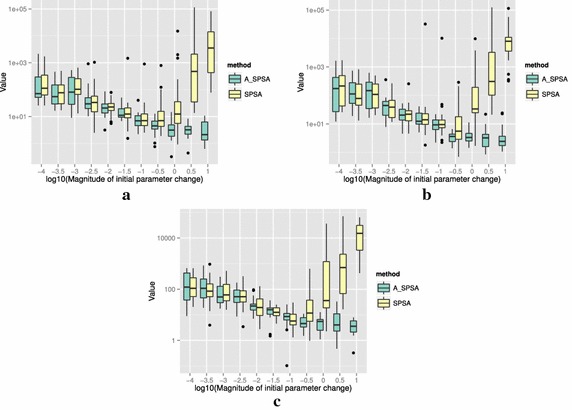
Initial parameter change $\delta \hat{\theta }_{0_{\min }}$ and distribution of responses after 2000 function evaluations for “Rotated Ellipsoid”. **a** No noise, **b** σ = 0.10, **c** σ = 1.0

For all the mathematical functions tested in this paper, optimization using SPSA diverges almost surely if the $\delta \hat{\theta }_{0_{\min }}$ is large. However, A_SPSA and SPSA give closely matching results when the initial step sizes are relatively small (i.e., the left hand side of the plots in Figs. 2, 3, 4, 5, 6, 7, 8, 9, 10 and 11). This is because, in cases that divergence does not happen, the adaptation of *a* does not take place in A_SPSA and therefore SPSA and A_SPSA have identical behavior. This is a confirmation that Algorithm 1 does not alter, in any significant way, the finite sample convergence characteristics of the original SPSA when the divergence does not manifest.

**Fig. 12:**
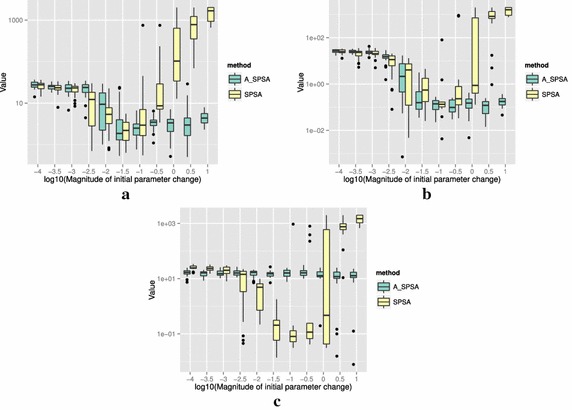
Effect of choice of *c* to the final response of “Sphere” with Gaussian noise of $\sigma = 0.1$ after 2000 function evaluations. **a** *c* = 0.01, **b** *c* = 0.10, **c** *c* = 1.00

**Fig. 13:**
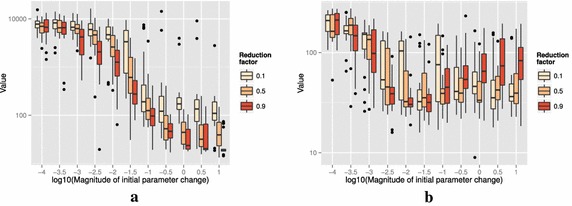
Effect of choice of the reduction factor of *a* to the responses after 2000 function evaluations. **a** Rosenbrock (no noise), **b** Rastrigin (no noise)

### Nonlinear dynamics example

We consider a parameter estimation problem with Lorenz attractor. Its nonlinear dynamics is described as

17 $$\begin{aligned} \frac{dx_1}{dt} = s(x_2-x_1), \end{aligned}$$

18 $$\begin{aligned} \frac{dx_2}{dt}= x_1(r-x_3)-x_2, \end{aligned}$$

19 $$\begin{aligned} \frac{dx_3}{dt}= x_1x_2-bx_1. \end{aligned}$$

We seek to identify the system parameters $\theta =[s,r,b]$ by minimizing the one-time-step-ahead prediction error $L_k$ of the state ${\mathbf {x}}_{k+1}$ given the current state ${\mathbf {x}}_k=[x_{k1},x_{k2},x_{k3}]^T$. We use fourth-order Runge–Kutta method to obtain ${\mathbf {x}}_{k+1}$.

Let us denote $\hat{{\mathbf {x}}}_{k+1}$ as one-time-step-ahead prediction given by the estimated system with parameters $\hat{\theta }_k$. Then, we can define the prediction error as

20 $$\begin{aligned} L_k ({\mathbf {x}}_k,\hat{\theta }_{k})= [{\mathbf {x}}_{k+1}-\hat{{\mathbf {x}}}_{k+1}]^T\cdot [{\mathbf {x}}_{k+1}-\hat{{\mathbf {x}}}_{k+1}]. \end{aligned}$$

Thus, the optimization to be solved is

21 $$\begin{aligned} \min _{\theta \in \varTheta } L_k({\mathbf {x}}_k,\theta ). \end{aligned}$$

The index *k* above is the same as the index *k* in the SPSA algorithms. So the SPSA iteration proceeds along with the time steps of the dynamic system to compute $L_k$.

We set the true parameters to be $\theta = [10, 28, 8/3]$ and pretend to not to know them. We set the time increment to be $\varDelta t = 0.005$ and simulate from $t = 0$ to 20, obtaining target state ${\mathbf {x}}_k$ with $k = 0,1,2,\ldots ,4000$. We let $\delta \hat{\theta }_{0_{\min }} \in \{0.001,0.01,1,10,100,1000\}$ and at each value of $\delta \hat{\theta }_{0_{\min }}$ we run both A_SPSA and SPSA 20 times.

For this problem, we set the parameter space as three-dimensional product space ${\varvec{\Theta }} = [0, 500]^3$. The initial state is ${\mathbf {x}}_0=[2,3,4]^T$. The initial guess (starting point) of the parameter set $\hat{\theta }_0$ is a random pick from ${\varvec{\Theta }}$.

**Fig. 14:**
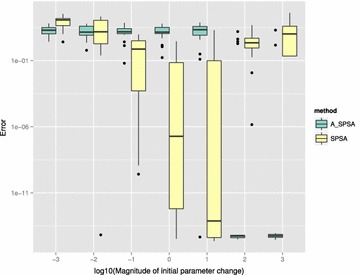
Initial parameter change $\delta \hat{\theta }_{0_{\min }}$ and distribution of $L_{4000}$ (after 8000 function evaluations)

Figure 14 show the box plots of final $L_k$ when started from different values of $\delta \hat{\theta }_{0_{\min }}$. The smallest median of final $L_k$ is obtained at $\delta \hat{\theta }_{0_{\min }}=10$ for SPSA and $\delta \hat{\theta }_{0_{\min }}=100$ and 1000 for A_SPSA. The best medians of final $L_k$ obtained for A_SPSA ($5.62\times 10^{-15}$) is smaller compared to that of SPSA ($3.10\times 10^{-13}$). However, both SPSA and A_SPSA had some runs that did not converge to the above mentioned near-zero $L_k$ values even at these $\delta \hat{\theta }_{0_{\min }}$.

Again, for A_SPSA, the best setting were obtained when $\delta \hat{\theta }_{0_{\min }}$ was set to large values near the order of magnitude of the distance between upper and lower bound of the domain, while for SPSA, the best $\delta \hat{\theta }_{0_{\min }}$ was at an interior value between $10^{-3}$ and $10^3$.

**Fig. 15:**
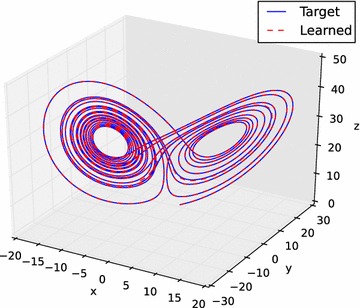
State evolution of the target and identified Lorenz attractor, $t=0$ to 20

Figure 15 shows the trajectory of the reference Lorenz attractor and the simulation of the Lorenz attractor whose system parameters *s*, *r*, and *b* were successfully identified by A_SPSA. The time *t* is run from 0 to 20 starting from the same initial condition used in the identification. The figure shows excellent match.

**Fig. 16:**
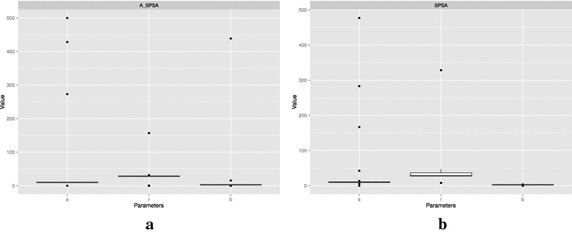
Distribution of the parameters identified by A_SPSA and SPSA. **a** A_SPSA with $\delta \hat{\theta }_{0_{\min }}=100$, **b** SPSA with $\delta \hat{\theta }_{0_{\min }}=10$

Figure 16 shows the box plots of parameters estimated by A_SPSA and SPSA starting at their best $\delta \hat{\theta }_{0_{\min }}$ settings. The corresponding statistics are shown in Tables 1 and 2. The boxes appear collapsed as single horizontal lines at medians since the spaces between first quartiles and third quartiles are very narrow. Some non-converging cases are visible as dots on the figure. The figure and the tables show that the parameter estimates are more consistent from run to run in A_SPSA than that of SPSA as A_SPSA has narrower first and third quartile differences.

**Table 1:** Statistics of identified Lorenz Attractor parameters by 20 SPSA runs at $\delta \hat{\theta }_{0_{\min }}=10$

	Method	*s*	*r*	*b*	Pred. Err. $L_{4000}$
1	A_SPSA: 0	Min.: 0.00	Min.: 8.017	Min.: 0.000	Min.: 0.0000
2	SPSA: 20	1st Qu.: 10.00	1st Qu.: 28.000	1st Qu.: 2.642	1st Qu.: 0.0000
3		Median: 10.00	Median: 28.000	Median: 2.667	Median: 0.0000
4		Mean: 55.94	Mean: 45.534	Mean: 2.311	Mean: 1.3645
5		3rd Qu.: 11.11	3rd Qu.: 36.817	3rd Qu.: 2.667	3rd Qu.: 0.1017
6		Max.: 477.04	Max.: 328.504	Max.: 3.261	Max.: 19.6773

**Table 2:** Statistics of identified Lorenz attractor parameters by 20 A_SPSA runs at $\delta \hat{\theta }_{0_{\min }}=100$

	Method	*s*	*r*	*b*	Pred. Err. $L_{4000}$
1	A_SPSA: 20	Min.: 0.000	Min.: 0.000	Min.: 0.0000	Min.: 0.0000
2	SPSA: 0	1st Qu.: 10.000	1st Qu.: 28.000	1st Qu.: 2.6667	1st Qu.: 0.0000
3		Median: 10.000	Median: 28.000	Median: 2.6667	Median: 0.0000
4		Mean: 68.069	Mean: 31.816	Mean: 24.8487	Mean: 1.2328
5		3rd Qu.: 10.000	3rd Qu.: 28.000	3rd Qu.: 2.6667	3rd Qu.: 0.0000
6		Max.: 500.000	Max.: 156.811	Max.: 438.8246	Max.: 15.6654

## Conclusion

With the adaptive initial step algorithm, one can avoid divergence in SPSA iterations. Moreover, with a large initial step size, the SPSA algorithm with the adaptive initial step algorithm was able to find equal or better solutions compared to the original SPSA for all the ten mathematical function minimization problems that we have tested. In the nonlinear dynamics example, the new algorithm was able to find system parameters more precisely. The proposed method may not eliminate the need of tuning the parameters of SPSA algorithms, but it facilitates the process by eliminating the risk of solution divergence and reducing the trial-and-error effort. Further testing of the algorithm with different test functions, noise distributions, and industrial use-cases would be beneficial. The improvement proposed in this paper is expected to be valuable when the objective functions are costly to evaluate or if the algorithm is employed inside another algorithm such as machine learning or target tracking, for manual tuning of the parameters would be cumbersome in such cases. As a future work, it would be beneficial to investigate under what conditions the probability of the proposed adaptation (i.e. going into if-branch in Algorithm 1) happening tends to zero as iteration *k* tends to infinity.
